# Neuropsychological Functions of *μ*- and *δ*-Opioid Systems

**DOI:** 10.1155/2013/674534

**Published:** 2013-12-26

**Authors:** Anna G. Polunina, Evgeny A. Bryun

**Affiliations:** Moscow Research and Practical Center for Narcology, Department of Healthcare, 37/1 Lyublinskaya ul., Moscow 109390, Russia

## Abstract

Brain opioid innervation is involved in many pathophysiological processes related to drug addiction. The main idea of the present review is that *μ*-/*δ*-opioid innervation is an intrinsic component of the motor/approach behavior network, which is activated synergetically with dopaminergic mesocorticolimbic network. Contribution of opioid innervation to the motor/approach behavior processing includes generation of positive emotions and inhibition of pain and stress reactions in order that the individual would be able to reach the vital goal. We cite the neuroanatomical data which showed that motor subcortical nuclei contain the most abundant opioid innervation and its activation is an obligatory component of positive emotions. In the majority of life situations, motor/approach behavior network concomitantly activates pain/stress control opioid network. Intensive cognitive activity induces activation of opioid innervation as well, and both enhancing and impairing effects of opioid agonists on cognitive functioning were demonstrated. Overall, the functioning of endogenous opioid networks may be summarized as following: NO physical/cognitive activity = NO positive emotions plus NO pain/stress control. We suppose that contemporary findings concerning neuropsychological functions of endogenous opioid system explain many controversial issues in neuropsychiatric conditions predisposing to drug addiction and neurological mechanisms of opioid addiction.

## 1. Introduction

Brain opioid innervation is intrinsically involved in many pathophysiological processes related to drug addiction. Multiple experimental studies showed that endogenous opioid system is not only the target of opioid addictive drugs but is also activated during alcohol and psychostimulants consumption [1, 2]. Moreover, genetic characteristics of endogenous brain opioid system are an important factor predisposing to drug abuse and addiction.

In addicted populations, the frequency of G allele of *μ*-opioid receptor is almost twice as high as in healthy populations [3, 4], and the efficacy of detoxification of alcoholic patients with G allele was shown to be lower in comparison with A/A genotype [5]. Ray and colleagues [6] observed higher levels of vigor and lower levels negative mood in alcoholics with Asp40 allele of the *μ*-opioid receptor gene in comparison with alcoholics who were homozygotes for the Asn40 variants. Association between cocaine addiction and polymorphism of *δ*-opioid receptor was shown in humans as well [7].

Insufficient activity of endogenous brain opioid systems appears to predispose to drug addiction. Learn and colleagues [8] showed that high-alcohol-drinking rats were characterized by lower density (10–30%) of *μ*-opioid receptors in hippocampus, thalamus, habenula, and amygdala in comparison with low-alcohol-drinking rats. Martín et al. [9] observed lower basal proenkephalin gene expression in striatum in rats with high vulnerability to morphine self-administration in comparison with animals with low motivation for morphine consumption. In humans with family history of alcoholism, diminished hypothalamic opioid tone was demonstrated as well [10].

Genetics is not the only regulator of activity of endogenous opioid systems. Sex steroids were shown to influence *μ*-opioid receptor trafficking and expression on plasma membrane [11]; overall females were consistently shown to express less *μ*- and *δ*-opioid receptors in cortex and brainstem in comparison with males [12, 13]. Interestingly, Vucetic and colleagues [14] demonstrated long-term effects of maternal diet during pregnancy and lactation on opioid receptor expression in an offspring. Expression of both *μ*-opioid receptor and preproenkephalin was increased in nucleus accumbens, prefrontal cortex, and hypothalamus of mice from dams that consumed high-fat diet during pregnancy and lactation. Finally, neonatal handling influenced the expression of *μ*-opioid receptors in the amygdalae, hippocampus, ventral tegmental area, nucleus accumens, and the prefrontal corex in the study of Kiosterakis et al. [15]. The researchers suggested that the increased ability to cope with stress and decreased emotionality in adult animals which were handled at neonatal period were mediated by increased expression of *μ*-opioid receptors after handling.

Dramatic and profound changes of brain opioid innervation after chronic use of pharmacological opioids are the main concern of medical specialists and lay public. Besides excruciating physical dependency symptoms (pain in low back and legs, autonomic hyperactivation, etc.), chronic morphine and heroin induce progressive personality distortions with acquisition of antisocial and criminal life-style in nearly all opioid addicts [16, 17]. Indeed, the opioid-induced personality changes appear to be more progressive and inevitable in comparison with alcohol and psychostimulant addiction [17–20]. Most important, opioid addiction is associated with high lethality. Only about 50% of these patients live longer than 20 years after an onset of opioid use [21], and about 10% of them attempt suicide during a 12-month period [22]. Hence, chronic abuse of pharmacological opioids impairs some existential neurobiological mechanisms which are an obligate component of normal personality functioning.

The recent studies showed that morphine interacts with opioid receptors differently in comparison with endogenous opioid ligands. In contrast to *μ*-opioid agonist DAMGO, morphine induces less prominent phosphorylation and internalization of *μ*-opioid receptors, and this mechanism may underlie development of tolerance to opioid effects in chronic morphine users [23, 24]. Groer and colleagues [24] observed complete reversal of DAMGO-induced phosphorylation of opioid receptors in 20 minutes after the removal of the drug. In contrast, morphine-induced phosphorylation of opioid receptors was both less robust and less reversible in comparison with DAMGO [24]. The data of Napier and Mitrovic [25] evidence that DAMGO and morphine may induce somewhat different neural and behavior responses. The authors observed potentiation of glutamate-evoked excitations after DAMGO injection into ventral pallidum, whereas morphine most often attenuated this effect. Hence effects of morphine-like substances do not absolutely resemble endogenous opioid system activity; nevertheless, pharmacological opioids interfere with activity of endogenous opioid systems, which controlling the vital neuropsychological and autonomic functions.

Brain opioid innervation includes three well-defined types of opioid receptors, and activation of either of them mediates analgesia [26–28]. At the same time, positive emotional effects of activation of *μ*- and *δ*-opioid receptors differ from aversive effect induced by *κ*-opioid agonists. The “classical” endogenous ligands of opioid receptors, that is, endorphins, enkephalins, and dynorphins, are peptides. The traditional opioid peptides share the common amino-terminal sequence of Tyr-Gly-Gly-Phe-(Met or Leu), which has been called the opioid motif [29]. *β*-endorphin is equiactive at *μ*- and *δ*-opioid receptors with much lower affinity for *κ*-receptors. [30]. Enkephalin has tenfold higher affinities for *δ*-receptors than for *μ*-receptors with negligible affinity for *κ*-receptors. Dynorphins have high affinity for *κ*-receptors and significant affinity for *μ*- and *δ*-receptors. Recently discovered endomorphins are highly selective for *μ*-receptors. The structures of endomorphin-1 and endomorphin-2 are quite distinct from those of the traditional opioid peptides [31]. Instead of the opioid motif at the N-terminus, endomorphin-1 is composed of two amino acid residues, Tyr and Trp, whereas in endomorphin-2, Trp is replaced by Phe.

Overall, direct effects of endogenous and pharmacological opioids are inhibitory; that is, activation of opioid receptors leads to hyperpolarization of postsynaptical or presynaptical neurons [32]. All of the opioid receptors are guanine nucleotide binding protein (G-protein) coupled receptors [28]. As with other members of the G-receptor family, each of the opioid receptors has been shown to inhibit high threshold voltage-activated calcium channels, that is, inhibiting neurons. Opioids are now known to activate a variety of potassium channels through G-protein activation and other mechanisms [28].

Inhibitory function of activated opioid receptors is well illustrated by studies of the role of opioids in the pathogenesis of epilepsies. Systemic naloxone facilitates epileptic activity in animals [33, 34]. In humans, the significant association between polymorphism of the *μ*-opioid receptor gene and idiopathic epilepsies was shown as well [35]. At the same time, opioids inhibit the release of inhibitory neurotransmition (e.g., GABA), and therefore many consequences of activation of opioid innervation are excitatory [28].

Knoll and Carlezon [36] noted that signaling mechanisms of neuropeptides including opioids differ from classical neurotransmitters (e.g., glutamate, GABA). Neuropeptides are released at both synaptic and extrasynaptic sites in response to sustained neuronal activity. Upon release, neuropeptides are more slowly degraded by extracellular peptidases and are therefore able to diffuse much greater distances. This mode of action enables neuropeptides to convey information and coordinate activity across broader networks of neurons [36].

In the present review, we purposed to summarize the present knowledge about neuropsychological functions of *μ*- and *δ*-opioid systems. We limited our focus on publications concerning *μ*- and *δ*-opioid innervation, as these two opioid subsystems are acting synergetically, whereas functions of *κ*-opioid innervations are somewhat different and are not as well studied as *μ*-/*δ*-opioid innervation is. In addition, we preferred to limit our scope by studies which used either endogenous opioid ligands or naloxone, whenever it was possible. However, some important issues have been studied only with application of morphine-like substances, and therefore we cited these studies as well.

The main idea of the present review is that *μ*-/*δ*-opioid innervation is an intrinsic component of the motor/approach network, which inhibits pain and stress reactions in order that the individual would be able to reach the vital goal. We cite the neuroanatomical data which showed that motor subcortical nuclei contain the most abundant opioid innervation, and its activation is an obligatory component of positive emotions. Although, we present data concerning motor/approach behavior and pain/stress control as separate neuropsychological functions of opioid innervation, this separation is clearly artificial, and in the majority of life situations, the motor/approach network concominantly activates pain/stress control network. Intensive cognitive activity induces pain/stress network as well, and we cite the data concerning opioid innervations effects on cognitive activity in the third chapter of the review.

## 2. Positive Emotions and Motor/Approach Network

The term “positive emotions” implies to be in a good mood, to feel optimistic, to feel satisfied with one's life, to experience well-being and happiness, and to consider that the quality of one's life is good [37]. Positive emotions are not only desirable, but essential aspects of the healthy personality. Deficit of positive emotions is a characteristic feature of depression [37]. Gross and Thompson [38] noted that positive emotions are commonly approach-related and manage appetitive behavior. Virtually all goal-directed behavior can be construed as maximizing pleasure or minimizing pain [38].

Subcortical nuclei involved into reward and motor/approach behavior processing are abundantly innervated by *μ*- and *δ*-opioid receptors, and most of these brain structures are the neuronodes of mesocorticolimbic dopamine network [39, 40]. Recently dopamine was considered to be the core mediator of reward processing; however, later studies evidenced that at least partially opioid rewarding effects are independent of dopamine innervation. At the same time, approach behavior network is widely recognized as a dopaminergic one; however, synergetic contribution of the opioid innervation to approach behavior patterns was clearly shown as well.

### 2.1. Ventral Tegmental Area (VTA) and Substantia Nigra

Ventral tegmental area and substantia nigra are the main sources of the dopamine innervation of subcortical nuclei and cortical mantle of the mammalian brain. Both structures are richly innervated by *μ*-opioid and to a lesser degree by *δ*-opioid receptors and at the same time, concentration of precursors of enkephalins and endorphins in brainstem dopamine nuclei is very low [1]. Recently, modest concentration of endomorphin-2 (natural selective *μ*-opioid ligand) was determined both in ventral tegmental area and substantia nigra [31]. The discrepancy between rich receptor innervation and low concentration of natural opioid receptor ligands in brainstem dopamine nuclei means that the opioid innervation in this region is under control of distant brain structures or global release of brain opioid peptides.

Experimental studies consistently showed that *μ*- and *δ*-opioid agonists are self-administered into the region of mesolimbic dopamine cell bodies of the VTA, and selective *μ*-agonists are more effective as rewarding agents in comparison with selective *δ*-opioids [39]. Injection of *μ*-opioids into this region induces activation of the dopamine system by inhibition of nearby GABAergic neurons, and this leads to burst firing of dopamine neurons [39].

Injection of opioid ligands into the substantia nigra does not induce reward, which is commonly measured as conditioned place preference or self-administration responses, but it is followed by topographically specific motor responses [41]. For instance, Bontempi and Sharp [41] reported that DAMGO injections into the laleral substantia nigra induced c-Fos in dorsolateral striatum and globus pallidus along with concomitant contralateral turning behavior. Interestingly, the length of turning behavior positively correlated with the dose of the opioid agonist ranging from 30 minutes to 2 hours.

Overall, the *μ*-opioid effects at VTA and dorsal dopamine nuclei are dopamine dependent and are followed by behavior activation. Dopamine neurons of VTA project to the ventral striatum, vetral pallidum, and anterior cingulate and orbitofronal cortex constituting the reward and approach behavior neurocircuitry [42, 43]. Whereas, projections of substantia nigra dopamine neurons reach dorsal striatum and dorsolateral prefrontal cortex, constituting motor and executive function networks.

### 2.2. Ventral Striatum, Dorsal Striatum, and Ventral Pallidum

Ventral striatum includes the nucleus accumbens, the ventral medial caudate, and the rostroventral putamen, whereas dorsal regions of these subcortical nuclei are defined as dorsal striatum [42]. Nucleus accumbens, caudate nucleus, and putamen are densely innervated by *μ*- and *δ*-opioid receptors [1, 30, 44], and concentration of opioid receptors in these structures is comparable only with opioid receptor density in thalamus and amygdale [45]. As opposite to brainstem dopamine nuclei, *μ*- and *δ*-opioid ligands (endomorphins and enkephalins) are highly concentrated in nucleus accumbens and striatum [31]. Moreover, striatum is the major source of enkephalin in the forebrain structures and globus pallidus, and approximately half of the spiny neurons of the caudate nucleus and putamen contain enkephalin as a coneurotransmitter [30].

Ventral pallidum is the primary output for the nucleus accumbens and other nuclei of ventral striatum, whereas globus pallidus receives major output from dorsal striatum [46]. These structures are densely innervated by *μ*-opioid and to a lesser degree *δ*-opioid receptors [1]. Moreover, the concentration of enkephalin in globus pallidus is the highest in comparison with other structures of the brain, but the source of pallidal enkephalin is the projections from striatum [30]. Neuroimaging studies consistently showed that ventral striatum and ventral pallidum are synergetically activated during reward processing [42].

Animals self-administer *μ*- and *δ*-opioid agonists to both nucleus accumbens and ventral pallidum [39, 46, 47]. Injections of *μ*- and *δ*-opioid agonists into the nucleus accumbens stimulate palatable food consumption [1].

Napier and Mitrovic [25] showed that local injections of opioids suppressed spontaneous firing of ventral pallidum neurons as opposite to the localized opioid effects at ventral tegmentum area. Nevertheless, *μ*-opioid receptor agonists potentiated the excitatory influences of cortical and amygdaloid glutamate innervation of ventral pallidum along with attenuating accumbal P-substance and GABA-ergic effects on neurons firing in this region. The authors suggested that opioid innervation of ventral pallidum is a cue mediator of the transduction of cognition and affect into behavior.

Smith and Berridge [40] showed that both nucleus accumbens and ventral pallidum contain *μ*-opioid hotspots supporting generation of “liking” (hedonic) affective condition as measured by the amplification of positive orofacial reactions. In their experimental studies, *μ*-opioid stimulation of the hedonic hotspots either in the nucleus accumbens or ventral pallidum generated increases in “liking” (increased liking) reactions for food reward, and the activation of *μ*-opioid receptors in one of these structures reciprocally recruited activation of Fos expression in the other one. At the same time, blockade of either of the hotspots by the naloxone prevented generation of “liking” reactions even when the other hotspot was stimulated. In contrast to “liking” reactions, “wanting” behavior (intake of the food) required only stimulation of the *μ*-opioid hotspot in the nucleus accumbens. Smith and Berridge [40] concluded that the *μ*-opioid hotspots in the nucleus accumbens and ventral pallidum interact cooperatively as a single opioid circuit to amplify the hedonic impacts of rewards.

It should be noted that eating behavior related opioid hotspots are only cubic-millimeter in volume in the rodent brain [48]. Moreover, Smith et al. [46] reported opioid “coldspots” in anterior regions of the ventral pallidal, in which *μ*-opioid stimulation abolished food reward and even caused sucrose aversion. Besides eating behavior, ventral pallidum controls affiliation, sex and pair bonding as well, and perhaps some other rewarding behaviors, and therefore activation of opioid system may be topographically specific for special forms of behavior. Smith and colleagues noted that eating behavior opioid hotspot in posterior ventral pallidum is characterized by higher enkephalin levels along with less dense concentration of presynaptic *μ*-opioid receptors in comparison with the anterior region of ventral pallidum. The authors suggested that opioid hotspot in the posterior pallidum is characterized by a basic level of tonic activation.

Although, *μ*- and *δ*-opioids are not self-administered into the dorsal striatum, *μ*- and *δ*-opioid innervation in this region is an important trigger for reward-related behaviors. Di Feliceantonia and colleagues [49] observed elevation >150% enkephalin surgers in anterior dorsomedial neostriatum in rats while consuming palatable chocolates in comparison with baseline condition. Moreover, the researchers observed intense >250% increase of intake of palatable sweet food without concomitant increase of “liking” reactions after injection of DAMGO into this region. This effect of *μ*-agonist was strictly localized to the anterior dorsomedial region of neostriatum and was absent during injections into the neighbourhood structures.

Nielsen and colleagues [50] showed involvement of *δ*-opioid receptors in the dorsal striatum into binge-like patterns of excessive ethanol drinking. The authors injected selective *δ*-opioid receptor antagonist (naltrindole) into dorsal striatum and observed reduction of ethanol consumption in young rats. In other study by Bontempi and sharp [41], it was shown that selective blockade of *μ*-opioid receptors in dorsal striatum, but not in nucleus accumbens, disturbed social attachment formation in monogamous animals, and the researchers concluded that *μ*-opioid innervations of caudate-putamen region are an obligatory component of adult affiliation neurocircuitry.

Although initial motor response of naïve animals to the injection of morphine may be decrease of motor activity (cataplexy), the common effects of *μ*- and *δ*-opioid agonists include increase of locomotor activity [31, 51]. Hence, opioid system potentiates motor activation induced by “motor” neuromediator dopamine [31]. Nevertheless, at the level of striatum, dopamine-potentiating and enhancing effects of *μ*-/*δ*-opioid ligands on reward and locomotor activity are due to inhibition of cholinergic interneurons, which tonically inhibit dopamine-sensitive neurons [1, 52, 53]. Interestingly, Jabourian and colleagues [52] showed that the quantity of striatum cholinergic interneurons expressing *μ*-opioid receptors is characterized by diurnal variation with the lowest number of neurons with opioid receptors in the morning (32%), whereas 80% of striatum cholinergic cells expressed *μ*-opioid receptors in the afternoon. The enkephalin release was the highest in the afternoon as well. Seasonal variations of the opioid system activity were also reported. Zuikov and colleagues [51] showed 9-time decrease of locomotor activity in gophers after naloxon injection in the autumn period, whereas in spring period effects of naloxone were much less prominent with only 2-time decrease of locomotor activity.

### 2.3. Limbic Cortex, Neocortex, and Amygdale

In humans, amigdala, anterior cingulate, and insular cortex are characterized by very high concentration of opioid receptors, which is similar to basal ganglia and is only a little bit lower in comparison with thalamus [45]. In other mammals, *μ*-opioid receptors are densely distributed in anterior cingulate cortex (especially perigenual cortex) and amygdale as well [1, 44, 54]. The distribution of *δ*-receptors is most abundant in layers 1 and 2 of the neocortex and amygdala [30]. In contrast to *μ*-receptors, *δ*-opioid receptors bind densely in the hippocampal formation, particularly in the dentate gyrus. Overall, *δ*-opioid receptors predominate in forebrain structures, such as neocortex and amygdala [44]. It is logical that enkephalin is the most abundant opioid agonist in anterior cingulate cortex, insular cortex, and amygdale [54, 55].

Neuroimaging studies of healthy subjects consistently showed activation of limbic cortex and subcortical structures after *μ*-opioid agonist injection, whereas sensory and dorsolateral prefrontal cortex tended to deactivate in this condition [56–59]. The activation of anterior cingulate cortex after *μ*-agonists was shown both in healthy subjects [57–59], and opioid addicts [60]. In the study of Khalili-Mahani et al. [59], the pregenual anterior cingulate cortex was one of the regions which showed the highest increase in absolute cerebral blood flow after morphine injection. Nevertheless, Becerra and colleagues [56] reported deactivation of anterior cingulate cortex after low doses of morphine.

Activation of insular cortex, operculum, and amygdala after systemic opioid agonists was demonstrated in several studies as well [57–59]. Orbitofrontal cortex was shown to be highly activated at least in two studies [56, 57]. In addition, Becerra and colleagues [56] observed activation of hippocampus, nucleus accumbens, putamen, hypothalamus, and substantia nigra after intravenous morphine injections to drug-naïve subjects. These effects gradually developed and were observed for about 20 minutes after injection. Only orbital gyri and hypothalamus showed rapid increase of activation. Importantly, in the study of Leppä et al. [57] activation of orbitofrontal, insular cortex, and amygdala, closely related to most subjective opioid effects (euphoria, calmness, analgesia, etc.).

At the same time, at least two neuroimaging studies reported bilateral decrease of activity in neocortex and some subcortical structures after *μ*-opioid agonist injection in healthy subjects. Becerra and colleagues [56] observed decrease in signal in the dorsolateral prefrontal cortex, temporal lobe, inferior parietal lobe, thalamus, and periaqueductal gray/ventral tegmentum. Similar decrease of cerebral blood flow in precentral gyrus, angular cortex, precuneus, temporooccipital, and frontoparietal regions was observed in the study of Khalili-Mahani et al. [59].

Petrovic and colleagues [61] studied effects of naloxon administration to healthy subjects before Gambling test. Under the influence of naloxone, subjects rated rewards as less pleasurable. In the loss condition, subjects rated negative outcomes as more aversive after naloxone compared with placebo, and this negative trend was related to enhanced activity in anterior insula and caudal anterior cingulated cortex after naloxone. In placebo condition, both larger rewards and larger losses were associated with enhanced activity in rostral region of anterior cingulate cortex; however, naloxone attenuated these effects of reinforcement magnitude both for losses and rewards. The authors suggested that the outcome magnitude-related activation in rostral ACC may be associated with opioid regulation of the hedonic experience in rewards and countering the aversive experience of losses. In addition, these data may indicate that caudal ACC region is involved into the hedonic evaluation of loss processing.

The balance between magnitudes of rewards and losses is the most important factor for decision making, and magnitude of losses is twice as important as magnitude of rewards in both humans and animals [62–64]. The cited data evidence that endogenous *μ*-/*δ*-opioid system shifts the magnitude of pleasurable and aversive outcomes into the positive direction and increase probability of active approach to positive stimulus and ignoring negative stimuli.

Kringelbach and Berridge [48] noted that cortical representations of hedonic values are coding rather than causing pleasure. Given the abundant projections of orbitofrontal and anterior cingulate cortex to nucleus accumbens and ventral pallidum, it seems to be possible that activation of cortical representation of complex object/event values induces activation of subcortical hedonic hotspots, which in turn “generate” pleasure condition.

The investigations of the role of rich opioid innervation of amygdale have only recently been started. Mahler and Berridge [65] showed that activation of *μ*-opioid neurotransmission in central nuclei of amygdale enhanced appetitive and consummatory behaviors directed toward each animal's own prepotent conditional stimulus, more than toward the alternative conditioned stimulus or other stimuli in the chamber. Appetitive and consummatory behaviors directed toward the prepotent conditioned stimulus or food were enhanced regardless of whether DAMGO was administered during learning or after learning. The authors concluded that opioid neurotransmission in central amygdale helps determining which environmental stimuli become most “wanted.”

### 2.4. Interaction between Dopaminergic and Opioid Innervations

For many years, mesolimbic dopamine system was considered as a central mechanism for reward processing. Later studies showed that reward includes two components: hedonic reaction (“liking”) and incentive salience (“wanting”), and dopamine is responsible only for incentive salience processing [66–68]. Hedonic reaction and incentive salience are commonly involved into the same behavioral response; however, separate processing of hedonic reactions and incentive salience are not uncommon as well. Berridge and Aldridge [68] pointed that “wanting” (incentive salience) is not a sensory pleasure as “liking” is. Incentive salience is a means to make decisions among different types of rewards. When a cue is attributed with incentive salience by mesolimbic brain systems, it causes that cue and its reward both to become momentarily more intensely attractive and sought. In addition, the researchers noted that incentive salience may be processed unconsciously in certain situations.

Mesolimbic dopamine system is activated not only during rewarding situations, but also during aversive and stressful ones. Bassareo and colleagues [69] showed that both positive (sucrose and chocolate) and aversive (quinine and NaCl solution) stimuli induced release of dopamine in nucleus accumbens core and prefrontal cortex in rats. At the same time, activation of dopamine neurotransmission in nucleus accumbens shell was related to the novelty of appetitive stimulus rather than hedonic reaction to it. Faure and colleagues [70] demonstrated association between activity of dopamine innervation in caudal shell of nucleus accumbens and fearful behavior, whereas activity of dopamine neuromediation in rostral shell was related to appetitive behavior. In humans, activation of nucleus accumbance was demonstrated during both gain and loss conditions in task with monetary reward; however, reward induced more prominent and sustained activation in nucleus accumbens in comparison with loss condition [71, 72].

Davidson and colleagues [73] pointed to the existence of two fundamental brain networks that underlie approach- and withdrawal-related emotion or certain forms of positive and negative affect. The approach system facilitates appetitive behavior and generates positive affect, such as the emotion occurring as an organism moves closer toward a desired goal. The withdrawal system, on the other hand, facilitates the withdrawal of an organism from sources of aversive stimulation and/or organizes appropriate responses to cues of threat. A range of neuroimaging studies showed lateralization of prefrontal cortex activity to left hemisphere during positive affective response and, oppositely, predominantly right-sided activation during the production of negative affect [73, 74].

Zald and colleagues [75] demonstrated predominantly left-sided activation of dopamine transmission in medial caudate nucleus during reward condition in healthy subjects. The researchers stressed the fact that dopamine system activation was observed only in a small portion of the striatum, suggesting topographical specificity in dopamine transmission. At the same time, other portions of the left striatum (putamen and anterolateral caudate) and left thalamus showed a reduction in dopamine transmission. Delgado et al. [71] also observed left-lateralized response to reward in left caudate, ventral striatum, and medial temporal region in healthy subjects. At least in opioid addicts, intravenous injection of *μ*-opioid agonist induces left-lateralized brain activation, which correlated with euphoric effect of the drug [60].

Interestingly, Hagelberg and colleagues [76] reported significant association between euphoria/cheerfulness and increased biding potential of D2/D3 dopamine receptor tracer in the left posterior cingulate cortex after alfentanil injection in healthy subjects. In addition, the researchers observed increased biding potential of D2/D3 dopamine receptor tracer in the medial frontal cortex, dorsolateral prefrontal cortex, superior temporal cortex, anterior cingulate cortex, medial thalamus, putamen, and caudate nucleus after alfentanil injection [76, 77]. The researchers interpreted these findings as reflecting reduced dopamine release after alfentanil injection. Nevertheless, *μ*- and *δ*-opioid innervation appears to be an essential neuromodulator of approach behavior network activity, and its effects are commonly synergetic to dopamine innervation.

## 3. Control of Pain and Negative Emotions

Control of pain and negative emotional responses are obligatory during active approaching to wanted stimulus. Otherwise, withdrawal system would be activated, and approach behavior network would be inhibited for avoidance of further painful stimulation. The cited below data evidence that opioid antinociception/analgesia is an intrinsic part of functioning of approach behavior network.

### 3.1. Pain Control Network

Opioids are an essential neuromediators in antinociception neuronetwork, and thalamus is characterized by the most abundant *μ*-opioid innervation in comparison with all other brain structures [1]. Other sensory relay and integrative nuclei, that is, nucleus tractus solitaries, spinal trigeminal nucleus and dorsal horns, and periaqueductal gray, are densely innervated by *μ*-opioid receptors as well [1, 44, 54]. Enkephalin neurons serve as local interneurons in the primary sensory receiving areas of the dorsal horn of the spinal cord and the spinal tract of the trigeminal nucleus [30]. Dense enkephalinergic innervation and endomorphins are characteristic for periaqueductal gray and locus coeruleus [1, 30, 31].

Inhibition of pain impulses at dorsal horns is induced by both spinal and supraspinal opioid innervation [30]. Overall, prevailing role of supraspinal antinociceptive control over segmental opioid release in the spinal cord was shown in a range of studies [78]. For instance, stimulation of periaqueductal grey nuclei was shown to activate descending antinociceptive mechanisms and inhibit pain impulses at dorsal horns [30].

Gear and colleagues [79] demonstrated that intra-accumbens injection of either opioid antagonist (naloxone) or dopamine antagonist (flupentixol) blocks injury-induced antinociception. The authors concluded that mesolimbic dopamine system responds to noxious and aversive stimuli and is tightly involved into pain modulation processes. Neuroimaging studies of placebo effects confirmed the intrinsic role of nucleus accumbens and mesolimbic dopamine system in pain modulation [80]. It was shown that placebo administration was associated with the activation of dopamine neurotransmission that was exclusively localized in mesolimbic dopaminergic terminal fields, ventral caudate, ventral putamen, and nucleus accumbens. The magnitude of dopamine activation in the nucleus accumbens was positively correlated with the magnitude of analgesia (the change of pain intensity ratings). Placebo-induced dopamine release in nucleus accumbens was positively correlated with the magnitude of endogenous opioid release in the nucleus accumbens, vetral putamen, amygdala, and insular and anterior cingulate cortex. Nonresponders to placebo showed deactivation of nucleus accumbens after placebo administration.

The cited above data evidence that not only opioid system activation induces activation of motor/approach dopamine system, the reverse relationship; that is, activation of motor/dopamine system induces activation of opioid analgesia, is true as well. Overall, it seems very probable that synergetic activity of motor/dopamine system and opioid analgesia are tightly interconnected physiological mechanisms. For instance, motor cortex stimulation is a valuable approach for pain relief, and recently it was shown that chronic motor cortex stimulation induces significant changes in opioid innervation of anterior middle cingulate cortex and periaqueductal gray, correlating with pain relief [81]. In addition, Mueller et al. [82] showed that lower opioid receptor availability in motor and premotor areas was associated with stronger perception of cold-related pain in healthy subjects. The same study showed significant association between lower opioid receptors availability in left inferior frontal, anterior cingulate, and insular cortex and higher cold pain-sensitivity. Finally, de Oliveira and colleagues [83] observed in an experimental study that acute forced and voluntary exercise stimulated both the expression of *μ*-opioid receptors and the release of *β*-endorphin and other endogenous opioid peptides. Intensive cognitive activity activates endogenous opioid innervation as well [84].

Genetic differences and hormonal status may considerably affect pain responsiveness. Females are characterized by significantly lower expression of *μ*-opioid receptors in comparison with males, and proestrus females are characterized by the lowest level of *μ*-opioid receptors [12, 85]. Morphine anesthesia effectiveness is significantly lower in females as well, and one study reported that females required 30% more morphine to reach the same level of analgesia as males [12, 86].

Tan and colleagues [87] found statistically significant association between *μ*-opioid receptor polymorphism and self-administered weight-adjusted morphine in females after caesarean delivery. *μ*-opioid receptor 118G homozygotes used more morphine and reported higher pain scores than 118A carriers. Bruehl et al. [88] showed significant association between *μ*-opioid receptor gene polymorphism and pain sensitivity as well.

Bruehl and colleagues [89] observed the association between greater pain-induced increases in *β*-endorphin concentration and significantly lower pain sensitivity in healthy subjects. The authors concluded that subjects with low *β*-endorphin release are characterized by the inability to produce significant analgesia in stress situations. It should be noted that the restless legs syndrome is traditionally suggested to be associated with deficits of endogenous opioids [90]. The recent postmortem study showed reductions of *β*-endorphin and met-enkephalin positive cells by 37.5% and 26.4%, respectively, in the thalamus in the restless legs patients in comparison with controls [91].

Patients with chronic peripheral neuropathic pain are characterized by lower opioid binding potential in both hemispheres, whereas patients with central poststoke pain demonstrated the opioid binding decrease in the contralateral hemisphere in the study of Maarrawi and colleagues [81]. Klega and colleagues [92] demonstrated similar contralateral decrease of opioid receptor binding potential in patients with complex regional pain syndrome. The latter patient group demonstrated opioid binding decrease predominantly in contralateral amygdala and parahippocampal gyri and increased opioid binding in contralateral prefrontal cortical areas. High pain rating index was associated with opioid binding decrease in midcingulate and ipsilateral temporal cortex. Overall, these data evidenced that patients with neuropathic pain are characterized by decreased opioid receptor availability.

Interestingly, Jones and colleagues [93] demonstrated significant increase of opioid receptor binding in patients with rheumatoid arthritis in dependence of pain state (in pain *versus* no pain). These changes in opioid binding were global; however, the most prominent dynamics were observed in the gyrus rectus (part of the orbitofrontal cortex), anterior insula, amygdala, and anterior putamen.

Hence, the pain is an important signal of some unfavorable or even dangerous changes in organism. Nevertheless, when stressful environment is to be overcome, pain may lead to considerable limitations of saving activities. Opioid antinociception is aimed to block pain in order that the individual could undertake measures for escaping further damaging and improving the environment. In this context, it is not surprising that opioid antinociception is tightly associated with motor system functioning and dopaminergic innervation.

### 3.2. Stress Response Regulation, Anxiolytic, and Antidepressant Function

Intrinsic involvement of *μ*-opioid innervation into stress response regulation was demonstrated in numerous studies. Recently, anxiolytic and antidepressant activity of *δ*-opioid agonists was found as well [94].

Normally, short-term stress induces activation of endogenous opioid systems in both humans and animals as measured by the increased pain tolerance immediately after stress [95, 96]. For instance, Bandura et al. [95] showed that failure to dissolve cognitive problems induces naloxone-sensitive hypalgesia in healthy humans.

In healthy subjects, smaller pain-induced *β*-endorphin increase is associated with higher anger-out scores (managing anger via direct physical or verbal expression) [89]. In addition, subjects with high anger-out scores demonstrated hyperalgesic responses in comparison with controls. Interestingly, Bruehl et al. [88] confirmed the positive association between anger-out score and pain ratings only in subjects with the wild-type *μ*-opioid receptor gene whereas the group with the A118G gene polymorphism was characterized by the inverse association; that is, lower anger-out subjects were most hyperalgesic. The authors concluded that trait anger-out may be due to impaired ability to elicit endogenous opioid release in response to noxious stimuli at least in subjects with the wild-type *μ*-opioid receptor gene.

A range of psychiatric conditions associated with low stress tolerability and emotion dysregulation were consistently shown to be characterized by insufficiency of endogenous *μ*-opioid activation. Bolderline personality disorder is considered now as a condition associated with dysregulation of the endogenous opioid system [97, 98]. This psychiatric disorder prevails in females and is characterized by affective burst-outs and impulsive behaviors, aggression, self-harm, and low stress tolerability. In the study of Bandelow and colleagues [97], patients with borderline personality disorder demonstrated significantly greater *μ*-opioid binding potential in the orbitofrontal cortex, caudate nuclei, left nucleus accumbens, and left amygdale at resting-state in comparison with controls. The insufficient baseline activation of *μ*-opioid system in borderline personality disorder patients paralleled with greater negative scores during sadness induction in comparison with controls.

In contrast to hypoactivity of *μ*-opioid system at baseline, induced sadness was associated with greater endogenous opioid system activation in the pregenual anterior cingulated cortex, left orbitofrontal cortex, left ventral pallidum, left amygdale, and left inferior temporal cortex in borderline personality disorder patients in comparison with normal controls [98]. At the same time, significantly greater deactivation of opioid neurotransmission in the left nucleus accumbens and the right hippocampus/parahippocampus during sadness condition was observed in borderline personality disorder patients in comparison with controls in the same study.

Bandelow and colleagues [97] suggested that self-destructive behaviors of borderline personality disorder patients may be explained by unconscious attempts to stimulate their endogenous opioid system regardless of the possible harmful consequences. In accordance with this suggestion, Stanley et al. [99] found low levels of *β*-endorphin and met-enkephalin in cerebrospinal fluid in patients with episodes of non-suicidal self-injurious behavior in comparison with psychiatric patients without history of self-injuries. Although all patients in this study had a history of at least one suicide attempt, patients with nonsuicidal self-injurious behavior reported significantly higher levels of depression and hopelessness in comparison with controls.

Opioid system abnormalities were reported in major depressive disorder patients as well. Kennedy and colleagues [100] observed significantly greater decrease in *μ*-opioid receptor binding potential in the left temporal cortex, anterior insular cortex, thalamus, ventral basal ganglia, amygdale, and periamygdalar cortex during sustained sadness condition (recall of negative autobiographic event) in patients with major depressive disorder in comparison with normal controls. Decrease of *μ*-opioid receptor binding potential in the left inferior temporal cortex correlated with negative affect ratings during sadness condition, whereas prominent reduction of the binding potential in the rostral cingulate cortex was characteristic for patients who did not respond to antidepressant treatment. Interestingly, response to citalopram treatment in major depressive disorder was shown to be associated with the *μ*-opioid receptor gene polymorphism in the study of Garriock et al. [101].

In contrast to depressive patients, normal subjects consistently demonstrated deactivation of *μ*-opioid neurotransmission during the sustained sadness condition (focusing on an autobiographical event associated with a profound feeling of sadness) [98, 100, 102]. In the study of Zubieta and colleagues [102], the deactivation of *μ*-opioid neurotransmission (increased *μ*-opioid receptor availability) was the most prominent in rostral anterior cingulate cortex, in left inferior temporal cortex, bilateral amygdalae, and ventral pallidum in healthy females. The magnitude of *μ*-opioid system deactivation in rostral anterior cingulate and right ventral pallidum during sadness correlated with the increase in negative affect ratings, and deactivation in ventral pallidum bilaterally, in left amygdala, in left insular cortex, and in the hypothalamus was associated with the reductions in positive affect ratings.

Significant association between lower activity of *μ*-opioid innervation and excessive activation of the left inferior temporal pole during presentation of aversive stimuli was observed by Liberzon et al. [103]. In addition, Bertoletti et al. [104] found that children (8–10 years old) carrying the OPRM1-G allele of the *μ*-opioid receptor gene demonstrated enhanced N170 amplitudes in response to facial anger expressions, which paralleled higher social withdrawal scores. The authors concluded that endogenous *μ*-opioid innervation in the limbic regions plays an inhibitory/anxiolytic role.

Anxiolytic and antidepressant properties of *δ*-opioid receptor agonists were demonstrated in a range of studies as well [105, 106]. For instance, Randall-Thompson and colleagues [105] showed that bilateral microinjections of the selective *δ*-opioid receptor agonist (DPDPE) into the central amygdala reduced anxiety-like behavior both at baseline and after a forced swim in adult rats. The authors concluded that the activation of the *δ*-opioid innervations in the central amygdala is important for regulating anxiety level. Nevertheless, Saitoh and Yamado [106] showed that *δ*-opioid receptor agonist inhibited the amygdalar subpopulation of neurons expressing *μ*-opioid receptors as well.

Overall, the abundant amygdalar opioid innervation is considered as a cue region of stress response and anxiety level regulation by many researchers [105, 107, 108]. Chieng and colleagues [107] showed that the *μ*-opioid receptor agonist (DAMGO) induced inhibition of 61% of cells in the central amygdala and completely blocked neurons projecting to the parabrachial nucleus. The researchers concluded that opioids can directly inhibit output from the amygdale by activating distinct subpopulations of opioid-sensitive neurons. Interestingly, Beckerman and Glass [109] showed that *μ*-opioid receptors are commonly coexpressed in the same dendrites in central amygdala as glutamate receptors do and that both opioid and glutamate receptors are frequently associated with common intracellular organelles. The researchers suggested that this close spatial relationship raises the possibility that these proteins can form large macromolecular complexes, through direct heterodimerization or by indirect linkage.

Studies of opioid receptor knockout animals showed that *δ*-receptor knockout animals differ from *μ*-receptor knockout ones [1]. *δ*-opioid receptor mutants showed increased anxiety levels and depressive-like behavior along with increased motor impulsivity, suggesting a facilitatory role of *δ* receptor activity on inhibitory controls. At the same time, *μ*-opioid receptor animals demonstrate decreased motivation to eat, reduced maternal attachment, and abnormally low reinforcing effects of opioid and nonopioid drugs of abuse [1].

Mice lacking *β*-endorphin or preproenkephalin knockout mice fail to demonstrate normal antinociceptive response to stress and are characterized by increased anxiety and depressive-like responses after stress in comparison with the wild-type controls [96, 110].

## 4. Cognition, Learning and Neuroplasticity

*δ*-opioid receptors are abundant in layers 1 and 2 of neocortex and in hippocampus; however, precise functional role of cortical opioid innervation remains unclear [1, 30]. Given the predominantly inhibitory function of endogenous opioid system, it is not surprising that systemic injection of pharmacological *μ*- and *δ*-agonists inhibits sensory and dorsolateral prefrontal cortex in humans [56–59]. In animals, it was shown that application of morphine or *β*-endorphin disrupted interneuron network gamma-oscillations in hippocampus, and this effect concerned predominantly long-range interneuron synchrony [111]. Whittington and colleagues [111] concluded that opioid agonists may disturb cognition via disruption of interneuron network oscillations. Interestingly, Rodefer and Nguyen [112] reported improvement of attention-set shifting in aged rats after naltrexone administration with no effect of naltrexone in young rats. The authors concluded that opioid-related mechanisms may underlie some types of cognitive dysfunction associated with aging processes.

Nevertheless, sedative effects of *μ*-/*δ*-opioids are prominent only in naïve subjects, whereas patients chronically using opioids do not demonstrate sedation signs after drug intake [113]. Moreover, a range of studies showed that endogenous opioid innervation is activated during intensive cognitive activity [84, 95] and enhances cognitive performance [114–116]. At least three studies showed enhancing effects of *μ*-/*δ*-opioid innervation on memory via modulation of attention-related responses and learning [114–116].

Chaves and colleagues [114] observed enhancing effect of novel experiences on performance in memory tests in untreated and placebo-treated healthy subjects. At the same time, administration of naltrexone blocked the enhancing effect of novel experience on memory, and this finding evidences that endogenous opioids are involved in cognitive processing. Experimental study of Holahan and colleagues [116] demonstrated enhancing effect of low DAMGO dose injections into dorsal striatum on spatial information acquisition. The researchers observed that animals after low DAMGO dose showed better retention of the platform location and enhanced ability to alter their search strategy in comparison with controls. At the same time, high dose of DAMGO produced impairments in cognitive performance compared to control group.

It is unclear if opioids may enhance cognitive performance directly or only via dopaminergic mechanisms. Neuroimaging studies consistently showed that motivational state and reward affect cognitive performance by activating cortical and subcortical structures. For instance, Pessoa and Engelmann [117] observed enhancing effect of reward on attention and selection of sensory information, which correlated with activation of the mesocorticolimbic dopamine network.

Chronic administration of pharmacological opioids induces profound neuroplastic changes in experimental studies [118–120]. Robinson and colleagues [119] reported increase of density of dendritic spines in orbital cortex along with decrease of spine density in nucleus accumbens shell and sensory and medial frontal cortex after chronic morphine treatment. Ballesteros-Yáñez et al. [120] also observed enlargement of basal dendritic arbors of prelimbic pyramidal neurons along with reduction in the size and branching complexity of the dendritic arbors of pyramidal cells in the motor cortex in rats chronically treated by morphine. In addition, inhibitory effect of chronic morphine on neurogenesis in dentate gyrus of hippocampus was shown in the study of Eisch et al. [121]. Obviously, that opioid-induced neuroplastic changes underlie addition and personality distortions in opioid addicts [122–124]. It should be noted, that the risk of opioid abuse in chronic pain patients is especially high in young age, when neuroplastic processes are especially active [125].

It is unclear if endogenous opioids may induce as profound neuroplastic changes as pharmacological opioids do. Few data evidence that intensive activation of endogenous opioid system induces long-term changes in personality and behavior. Yim and colleagues [126] found that postpartum depressive symptoms in euthymic women developed three times more often in cases with high *β*-endorphin levels throughout pregnancy in comparison with controls; that is, excessive activation of *μ*-opioid system during pregnancy induced psychological symptoms of “opioid abstinence” after delivery. In experimental study of Burkett and colleagues [127], obligatory involvement of *μ*-opioid activation into the partner preference formation in monogamous prairie voles was shown as well.

## 5. Limitations

The present overview of neuropsychological functions of *μ*- and *δ*-opioid systems is completed by clinicians (Anna G. Polunina, neurologist, and Evgeny A. Bryun, narcologist), and therefore it was purposed to summarize the most psychiatry relevant information concerning global involvement of endogenous opioid systems into personality functioning. More detailed, biologically focused and systematic consideration of functions of brain opioid systems would be important in further analytic studies in this field.

## 6. Conclusion

Opioid innervation is an intrinsic component of the motor/approach behavior network, which is activated synergetically with dopaminergic mesocorticolimbic network. Contribution of opioid innervation to the motor/approach behavior processing includes generation of positive emotions and inhibition of pain and stress reactions in order that the individual would be able to reach the vital goal. Motor subcortical nuclei contain the most abundant opioid innervation, and its activation is an obligatory component of positive emotions. A range of studies evidenced that motor/approach behavior network concominantly activated pain/stress control opioid network. Intensive cognitive activity induces activation of opioid innervation as well, and both enhancing and impairing effects of opioid agonists on cognitive functioning were demonstrated. Overall, functioning endogenous opioid networks may be summarized as follows: NO physical/cognitive activity = NO positive emotions plus NO pain/stress control. Finally, opioid innervation is involved in memory and neuroplasticity processes, and this function of endogenous opioid system is of importance in the contex of addiction research.
